# Nrf2-Heme Oxygenase-1 Attenuates High-Glucose-Induced Epithelial-to-Mesenchymal Transition of Renal Tubule Cells by Inhibiting ROS-Mediated PI3K/Akt/GSK-3*β* Signaling

**DOI:** 10.1155/2019/2510105

**Published:** 2019-08-06

**Authors:** Jong Ho Shin, Kyeong Min Kim, Jin Uk Jeong, Jae Min Shin, Ju Hyung Kang, Kitae Bang, Joo-Heon Kim

**Affiliations:** ^1^Division of Nephrology, Eulji University College of Medicine, Daejeon, Republic of Korea; ^2^Division of Pathology, Eulji University College of Medicine, Daejeon, Republic of Korea; ^3^Division of Pediatrics, Eulji University College of Medicine, Daejeon, Republic of Korea

## Abstract

**Background:**

Epithelial-to-mesenchymal transition (EMT) is thought to play a significant role in the advancement to chronic kidney disease and contributes to the deposition of extracellular matrix proteins and renal fibrosis relating to diabetic nephropathy.

**Method:**

We studied the effect of Nrf2-HO-1 signaling on high-glucose- (HG-) induced EMT in normal human tubular epithelial cells, that is, HK2 cells. In short, we treated HK2 cells with HG and sulforaphane (SFN) as an Nrf2 activator. EMT was evaluated by the expression activity of the epithelial marker E-cadherin and mesenchymal markers such as vimentin and fibronectin.

**Results:**

Exposure of HK2 cells to HG (60 mM) activated the expression of vimentin and fibronectin but decreased E-cadherin. Treatment of HK2 cells with SFN caused HG-induced attenuation in EMT markers with activated Nrf2-HO-1. We found that SFN decreased HG-induced production of reactive oxygen species (ROS), phosphorylation of PI3K/Akt at serine 473, and inhibitory phosphorylation of serine/threonine kinase glycogen synthase kinase-3*β* (GSK-3*β*) at serine 9. Subsequently, these signaling led to the downregulation of the Snail-1 transcriptional factor and the recovery of E-cadherin.

**Conclusion:**

The present study suggests that Nrf2-HO-1 signaling has an inhibitory role in the regulation of EMT through the modulation of ROS-mediated PI3K/Akt/GSK-3*β* activity, highlighting Nrf2-HO-1 and GSK-3*β* as potential therapeutic targets in diabetic nephropathy.

## 1. Introduction

Diabetic nephropathy (DN) is not only one of the most serious complications in people with diabetes but also a leading cause of chronic kidney disease. The various histopathological lesions that are associated with DN are collectively described as fibrosis [1, 2]. Renal fibrosis is characterized by glomerulosclerosis, tubular atrophy, peritubular capillary loss, interstitial fibrosis, and chronic inflammation. Different insults such as hyperglycemia, epithelial damage, persistent inflammation, or progressive vascular loss can cause fibrosis [3].

Understanding renal fibrosis is critical to the development of therapies for DN. The pathogenesis of renal fibrosis is the abnormal activation and growth of renal myofibroblasts and the marked accumulation of extracellular matrix proteins [1, 4, 5]. It seems that renal fibrosis has special pathways, because mesangial cells, erythropoietin-producing cells, and tubular epithelial cells exist only in the kidney and are involved together in developing renal fibrosis. Among the systems modulating this pathologic progress, EMT is thought to play a pivotal role in progressive renal fibrosis and in the subsequent functional deterioration of the kidney [1, 2, 4, 6–8]. EMT is characterized by the acquisition of mesenchymal properties by epithelial cells, preferentially podocytes and proximal tubular cells, and is suggested as an important source of myofibroblasts in DN. HO-1 [9, 10], Notch [8, 11], EGFR [12], transforming growth factor-*β*1 (TGF-*β*) [7, 13], Wnt [5, 8], and Hedgehog [14] signaling have come into focus as not only key regulatory molecules but also targets for treatment of EMT and renal fibrosis.

Nuclear factor erythroid-derived 2-related factor 2 (Nrf2), a redox-sensitive transcription factor, regulates the expression of some antioxidant enzymes through the antioxidant response element (ARE) [15, 16]. Under oxidative and electrophilic stress conditions, Nrf2 and Kelch-like ECH-associated protein 1 (Keap1) complex were disrupted through modifying cysteine residues of Keap1, resulting in the activation of ARE-responsive genes within the nucleus. Sulforaphane (1-isothiocyanate-4-methylsulfinyl butane (SFN)), known as an activator of Nrf2, induces a modification in the cysteine thiols of Keap1 allowing Nrf2 nuclear translocation [17, 18]. Several studies have presented that SFN alleviated DM-related cardiac and endothelial damage [19–23]. Shang et al. [24] announced that SFN reduced GSK-3*β* phosphorylation in rat mesangial cells and ameliorated experimental DN via the GSK-3*β*/Fyn/Nrf2 signaling pathway. However, the mechanism by which SFN attenuates high-glucose-induced EMT is not yet clear.

To determine the impact of Nrf2-HO-1 on diabetic nephropathy, EMT was induced in human renal proximal tubule cells and its biological role in regulation of EMT was investigated.

## 2. Materials and Methods

### 2.1. Cell Line and Treatment

The immortalized human proximal renal tubule HK2 cell line (catalogue number 22190) was purchased from ATCC (USA). The cells were maintained in medium supplemented with 10% fetal bovine serum and 1% antibiotic-antimycotics (Gibco, Paisley, UK). After the cells were grown to subconfluence, the cells were treated at the same time with different concentrations of D-glucose (5 mM, 60 mM) for 1, 2, 4, 8, 24, 48, and 72 h, respectively. SFN treatment was performed at 0.63 *μ*m for 1 h prior to D-glucose stimulation.

### 2.2. Antibodies and Western Blot Analysis

The primary antibodies anti-*β*-catenin, anti-Akt, anti-phospho-Akt, anti-GSK-3*β*, anti-phospho-GSK-3*β*, anti-snail-1, anti-slug, and anti-twist were acquired from Cell Signaling Technology (Beverly). The anti-E-cadherin, anti-fibronectin, and anti-vimentin antibodies were purchased from Dako (Glostrup, Denmark). The cells were lysed with cell lysis buffer on ice for 30 min. The cell lysate was resolved by SDS-PAGE on 10 or 12% polyacrylamide gels and transferred to PVDF membranes. The membranes were incubated with the appropriate primary antibodies for 2 h at room temperature.

### 2.3. Growth Study

The HK2 cells were seeded at a density of 2 × 10^5^ per well in 6-well plates and cultured for three days after which they were treated with different levels of glucose concentrations. Cells were then trypsinized, stained with 0.4% trypan blue (Invitrogen), and counted daily using a hemocytometer (Fisher Scientific, Allentown, PA, USA).

### 2.4. Measurement of Intracellular ROS

The intracellular ROS levels were detected by flow cytometry using the ROS-specific fluorescence probes 5- (and 6-) chloromethyl-2′,7′-dichlorodihydrofluorescein diacetate, acetyl ester (DCF-DA) (C6827). Finally, the intracellular ROS levels and their analyses were done using a Cytomics FC 500 flow cytometer (Beckman Coulter, Miami, FL) with Cytomics RXP and MultiCycler software.

### 2.5. Plasmid Vector Construction and Transfection of Ad-HO-1 Gene

The following HO-1 gene-specific primer set for PCR was used: 50-ATG AAT TCA TGG AGC GTC CGC AAC CCG-30 (sense) and 50-ATC TCG AGT CAC ATG GCA TAA AGC CC-30 (antisense). The HO-1 gene was inserted into the pcDNA3 vector (Invitrogen, CA, USA) by EcoRI and XhoI, and the HA epitope was tagged using KpnI and EcoRI. In brief, the gene of HA-tagged HO-1 was cut out from pcDNA3/HA-HO-1 by KpnI and XbaI enzymes and inserted into pAdTrack-CMV. Recombinant viral plasmids were amplified in HEK293 cells. HK2 cells were placed into 6-well culture plates incubated at 37°C for 24 hours at a cell density of approximately 1.6 × 10^5^ cells/well. When the cells reached 80% to 90% confluence, the adenovirus was added into each well (MOI = 80, 1.3 *μ*L Ad-HO-1).

### 2.6. Statistical Analysis

The data are shown as the mean ± SD of *n* determinations unless noted otherwise, and Student's test was used to compare two groups. Probability values (*p* < 0.05) were regarded statistically significant.

## 3. Results

### 3.1. Induction of EMT by HG in Normal HK2 Cells

**Figure 1:**
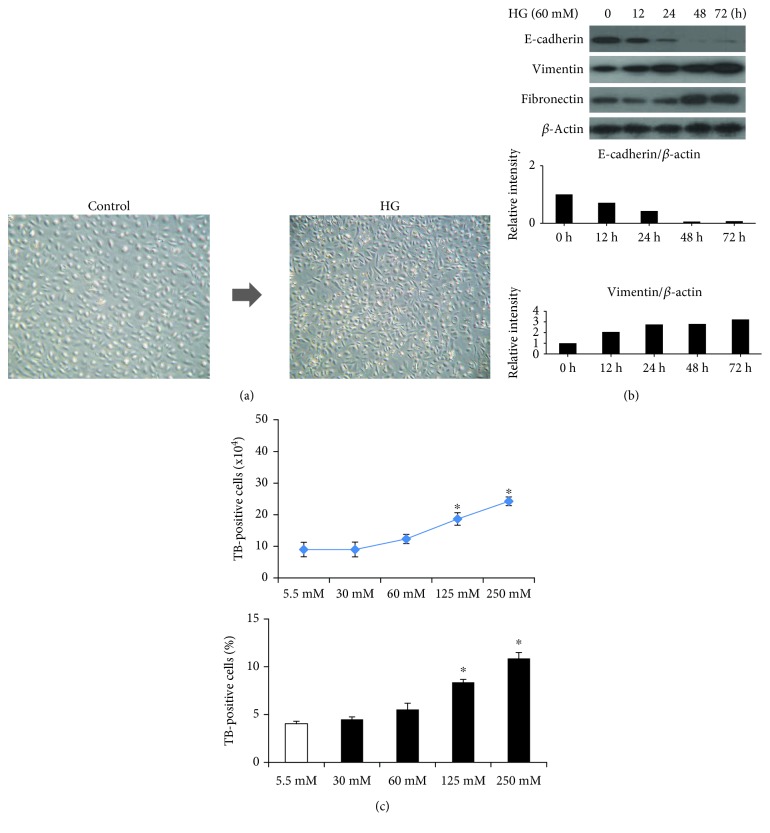
HG-induced EMT: a change of morphologic phenotype in human HK2 cells. Representative photomicrograph shows round- to oval-shaped growth in normal renal tubular cells, which converted into bipolar- or spindle-shaped cells with a fibroblast-like morphology after continuous stimulation with high glucose (60 mM/L D-glucose) for 72 h (right ×200, left ×100). (a) Exposure of HK2 cells to HG decreased the protein expression of the epithelial cell marker E-cadherin and increased the expression of the mesenchymal markers vimentin and fibronectin. (b) Cell viability exclusion assay by trypan blue staining. The number of trypan blue-positive cells was increased at a concentration-dependent manner in HG-treated HK2 cells (c).

We observed a change of morphologic phenotype in HK2 cells after HG (60 mM/L D-glucose) treatment was done. The HK2 cells showed round- to oval-shaped growth in normal conditions, which converted into spindle-shaped cells with a myofibroblast-like phenotype after continuous stimulation with HG for 3 days (Figure 1(a)). In order to evaluate the pathologic mechanism of EMT by HG, we observed the expression pattern of vimentin, E-cadherin, and fibronectin. Exposure of HK2 cells to HG for 12~72 hours inhibited the protein expression of the characteristic epithelial adhesion molecule E-cadherin and increased the expression of the myofibroblastic mesenchymal markers fibronectin and vimentin (Figure 1(b)). L-Glucose did not change any of these phenotypes, which suggested that not high osmolarity but HG per se induced EMT of HK2 cells. To determine cell viability (toxicity) of HG on HK2 cells, we performed the trypan blue exclusion assay. The number of trypan blue-positive cells was increased depending on HG concentration (Figure 1(c)).

### 3.2. Involvement of PI3K/Akt and GSK-3*β* Signaling on HG-Induced EMT of HK2 Cells

**Figure 2:**
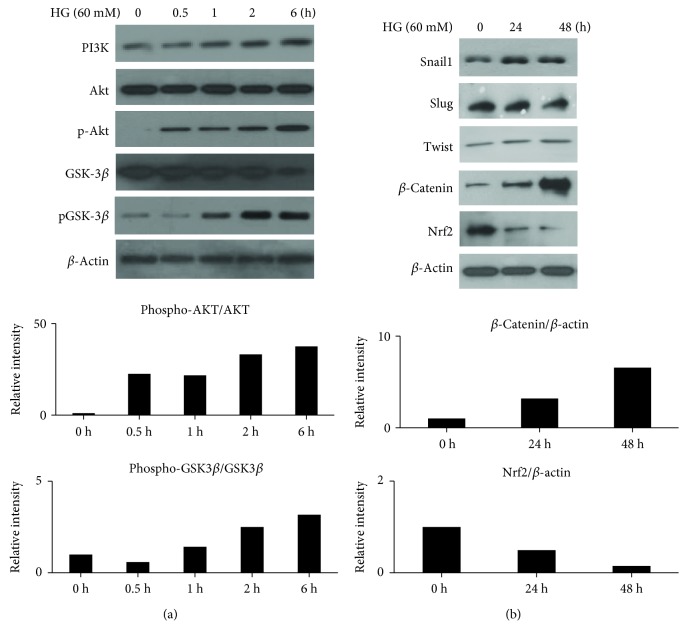
HG induced the phosphorylation of the receptor tyrosine kinase of human HK2 cells. HK2 cells were treated with HG for 24 h and then subjected to western blotting assay. Representative blots of the total protein levels of PI3K, the phosphorylated Akt, and GSK-3*β* (a). The inhibitory phosphorylation of GSK-3*β* increased Snail-1 and *β*-catenin protein levels. The protein level of Nrf2 was decreased (b).

To investigate the molecular mechanism of the EMT process induced by HG, we assessed the activity of the PI3K/Akt signaling after HG treatment for different periods of time in HK2 cells. HG induced a fast activation of PI3K/Akt and augmented phosphorylation inhibition of GSK-3*β* activity in the HK2 cells (Figure 2(a)). We next sought to investigate whether GSK-3*β* activity induced by HG involved the regulatory process of EMT-related genes such as Snail-1, slug, twist, and *β*-catenin. As expected, the treatment of HK2 cells with HG increased Snail-1, but not slug and twist, and then it increased *β*-catenin expression (Figure 2(b)). The exposure of HK2 cells to HG decreased the protein expression of Nrf2. These results indicate that PI3K/Akt/GSK-3*β* signaling induces the upregulation of Snail-1 and *β*-catenin activity which is translocated into the nucleus and interacts with transcription factors of the T cell-specific transcription factor (TCF), which contributes to the HG-induced EMT process in HK2 cells.

### 3.3. Inhibitory Effect of Nrf2-HO-1 on HG-Induced EMT in HK2 Cells

**Figure 3:**
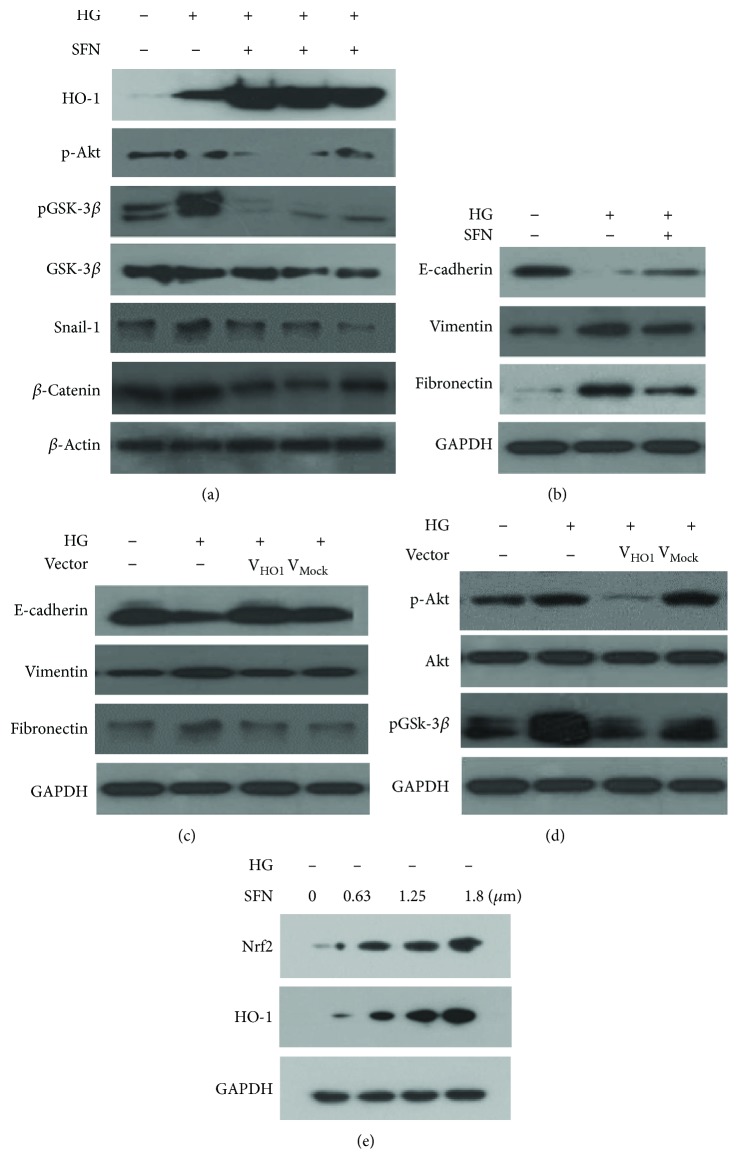
SFN treatment positively regulated HO-1 expression level and decreased the phosphorylation of PI3K/Akt473. Consequently, the inhibitory phosphorylation of GSK-3*β* was reduced and then Snail-1 and *β*-catenin decreased (a). EMT modulation by SFN. SFN treatment resulted in a decrease of the expression of vimentin and fibronectin and a recovery of the expression of E-cadherin (b). EMT modulation by HO-1 adenovirus transfection (c), similar to the reaction by SFN; it was also shown by HO-1 adenovirus transfection (d). The Nrf2 activator was treated with 0.63, 1.25, and 1.8 *μ*m of SFN to determine the adequate drug concentration for EMT induction in human HK2 cells (e). Western blot analysis showed that Nrf2 and HO-1 proteins were detected as a single band of about 48 kDa and 124 kDa, respectively, at a concentration-dependent manner.

We hypothesized that the Nrf2 pathway could be associated with the HG-induced EMT process because we previously observed that HO-1 or the HO-1 agonist attenuated HG-induced EMT in human mesothelial cells. HK2 cells basally expressed HO-1, as shown in Figure 3(a). HK2 cells were treated with the Nrf2 activator SFN and HO-1 adenovirus to elucidate the regulatory role of HG-induced EMT signaling pathways. SFN increased Nrf2-HO-1 protein levels and significantly reversed the HG-induced protein activities of PI3K/Akt and the inhibitory phosphorylation of GSK-3*β*, resulting in a decreased expression of Snail-1 and *β*-catenin in response to HG (Figure 3(a)). Moreover, SFN blocked the HG effects of decreased E-cadherin expression and increased vimentin and fibronectin expression by Western blotting (Figure 3(b)). The HO-1 overexpression by HO-1 adenovirus transfection reversed the HG-induced decrease in E-cadherin and increase in vimentin and fibronectin expression (Figure 3(c)). HO-1 adenovirus transfection induced a response similar to that by SFN (Figure 3(d)).  Figure 3(e) shows that the expression of HO-1 was stimulated by SFN. HO-1 adenovirus-transfected HK2 cells showed a well-reserved round to ovoid cell morphology, but Mock-HO-1 control showed spindle-shaped cells.

### 3.4. Involvement of ROS in HG-Induced EMT of HK2 Cells

**Figure 4:**
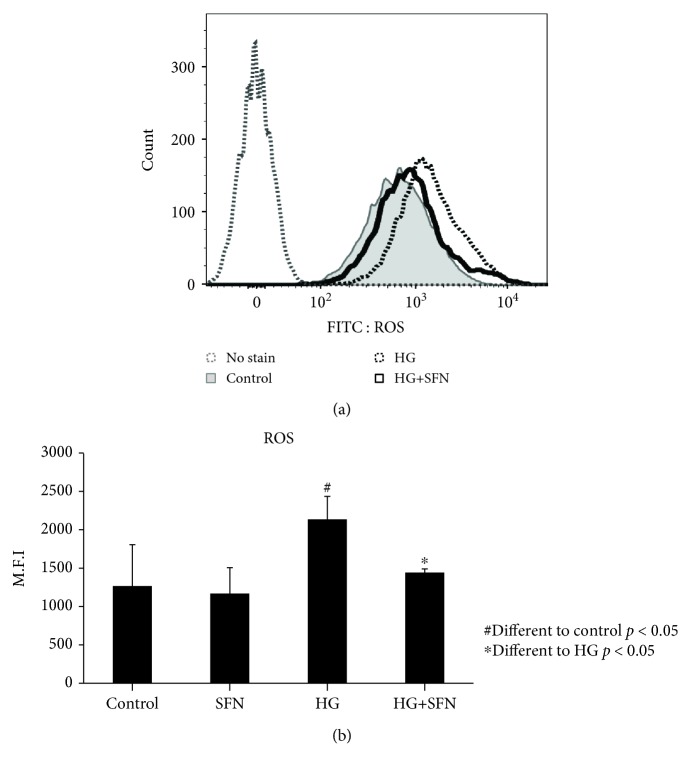
ROS regulation in human HK2 cells by SFN under high-glucose stress (assay by FACScan). High glucose induces an increase of intracytoplasmic ROS in human HK2 cells, but SFN treatment shows a left-shift pattern of the ROS curve.

To investigate how ROS is involved in the HG-induced EMT process in renal tubular epithelial cells, we measured intracellular ROS levels using DCF. When we treated HG in HK2 cells, the DCF-sensitive intracellular ROS level was increased, accompanied by HG-induced activation of PI3K/Akt. But a 12-hour pretreatment with the Nrf2 activator SFN blocked this increase in ROS and consequently decreased the activation of PI3K/Akt/GSK-3*β* signaling and EMT (Figures 4(a) and 4(b)). This result suggests that ROS may be importantly involved as an initial event in HG-induced EMT.

## 4. Discussion

DN is a long-standing complication of diabetes mellitus and currently the most common cause of chronic kidney disease. Recent studies have shown that the inflammatory process is related to the initiation and progression of diabetic nephropathy [25, 26]. Lots of stimuli, including hyperglycemia, glycation end products, and ROS induce the functional and structural changes [3]. In this study, we confirmed that HG increases the DCF-sensitive intracellular ROS levels, followed by the activation of PI3K/Akt in HK2 cells. This result suggests that ROS may be importantly involved as an initial event in HG-induced EMT and renal fibrosis.

EMT is a transdifferentiation process involving tissue remodeling associated with diabetic nephropathy in which transcription program switching can be induced by a variety of signaling pathways including receptor tyrosine kinases, TGF-*β*, and Wnt. When HG was treated in HK2 cells, phosphorylation activation of PI3K and Akt by HG-mediated ROS induced an inhibitory phosphorylation of GSK-3*β*, which was involved in the EMT-related signaling pathway. This result suggests that ROS-mediated PI3K/Akt/GSK-3*β* signaling may influence cellular dysfunction and the secondary adaptation in the renal tubule cells. Therefore, effective agents blocking the source of ROS production may hold the promise to protect the kidney from oxidative damage and prevent subsequent progression of renal fibrosis. Increased oxidative stress may result from the overproduction of ROS in the context of concomitant or insufficient antioxidant pathways. We observed that intracellular Nrf2 was decreased when HK2 cells were treated with HG. The reason why Nrf2 protein expression is decreased may be that the expression of cytosolic Nrf2 is decreased in HG concentrations, accompanied by the decreased number of the cuboidal-shaped cells. The expression of cytosolic Nrf2 was increased in SFN-treated HK2 cells and resulted in the reacquisition of the epithelial phenotype (data not shown). A treatment with the Nrf2 activator also blocked an increase of ROS and consequently decreased HG-induced activation of the PI3K/Akt signaling pathway and inhibitory phosphorylation of GSK-3*β*. Nrf2 and heme oxygenase-1 (HO-1), a downstream target gene of Nrf2, play a vital role in scavenging ROS and attenuating oxidative stress. In experiments to confirm the therapeutic effect of Nrf2-HO-1 signaling in HG-induced EMT, pretreatment with the HO-1 overexpression adenovirus vector reversed the HG-induced decrease in E-cadherin and increase in vimentin or fibronectin expression. Here, ectopic overexpression of HO-1 showed a change of the cellular morphologic phenotype in HG-induced mesenchymal phenotype cells with loosening intercellular contacts. The results suggest that the Nrf2-HO-1 pathway attenuates HG-induced intracellular ROS and prevents the progression of EMT.

The GSK-3*β*, which is a ubiquitously expressed serine/threonine kinase, is necessary to maintain the epithelial architecture [27] and can be inactivated by various signaling mechanisms, including the Wnt [28], lipid kinase PI3K/Akt [29], and extracellular signal-regulated kinase-1/2 (ERK-1/2) MAPK [30] pathways. In addition, GSK-3*β* activity can be regulated by oxidative stress and excessive extracellular material deposition, and it contributes to the pathogenesis of DN [31–33]. In our study, we observed that modulation of GSK-3*β* activity by SFN decreased EMT-regulatory gene Snail-1 and *β*-catenin expression in the HG-induced EMT experimental model of HK2 cells. Consequently, HG-treated HK2 cells showed a recovery of E-cadherin expression and decreased expression of mesenchymal markers. This result suggests that GSK-3*β*/*β*-catenin regulatory activities by Nrf2-HO-1 would be important in providing a novel strategy for the treatment of renal fibrosis and DN.

In conclusion, this study provides evidence that the Nrf2-HO-1 system exerts its anti-EMT effect on renal tubule cells through the inhibition of the PI3K/Akt/GSK-3*β* signaling pathway. We showed that SFN decreased the intracellular ROS level induced by HG and inhibited the phosphorylation of PI3K-Akt, and then it modulated GSK-3*β*/*β*-catenin activities. Consequently, these signaling activities resulted in the downregulation of the Snail-1 transcriptional factorand the recovery of E-cadherin. Therefore, our results suggest that the therapeutic targeting for Nrf2-HO-1 signaling attenuates the progression of renal fibrosis associated with diabetic nephropathy, which is manifested by the PI3K/Akt/GSK-3*β* pathway.

## 5. Conclusion

### 5.1. Strengths

Taken together, we showed that treatment of HK2 cells with SFN showed an amelioration of HG-induced changes in markers of EMT with an increase of HO-1 expression in experimental diabetic nephropathy. Our results suggest that Nrf2-HO-1 has a critical role in the regulation of HG-induced EMT through the modulation of the PI3K/Akt/GSK-3*β* activity, highlighting Nrf2-HO-1 as a potential therapeutic target in diabetes-induced nephropathy.

### 5.2. Limitations

Further studies are warranted to elucidate the signaling mechanism of diabetes-induced nephropathy.

## Data Availability

The data used to support the findings of this study are available from the corresponding author upon request.

## Abbreviations

EMT: Epithelial-to-mesenchymal transition
DN: Diabetic nephropathy
Nrf2: Nuclear factor erythroid-derived 2-related factor 2
ROS: Reactive oxygen species
SFN: Sulforaphane
HO-1: Heme oxygenase 1
HK2: Human kidney 2.
